# Visual standardized quantification of late gadolinium enhancement, a contour-less method for late gadolinium enhancement quantification

**DOI:** 10.1093/ehjimp/qyag148

**Published:** 2026-08-26

**Authors:** Giovanni Donato Aquaro, Roberto Licordari, Carmelo De Gori, Giancarlo Todiere, Umberto Ianni, Andrea Barison, Antonio De Luca, Alessandro Folgheraiter, Crysanthos Grigoratos, Mattia Alberti, Lisa Fulceri, Marilena Lombardo, Raffaele De Caterina, Gianfranco Sinagra, Michele Emdin, Gianluca Di Bella

**Affiliations:** Department of Surgical, Medical and Molecular Pathology and of Critical Area, University of Pisa, Via Savi 10, 56126 Pisa, Italy; Department of Clinical and Experimental Medicine, University of Messina, Messina, Italy; Fondazione Toscana G. Monasterio, Pisa, Italy; Fondazione Toscana G. Monasterio, Pisa, Italy; Cardiology Unit, San Benedetto del Tronto, San Benedetto del Tronto, Italy; Fondazione Toscana G. Monasterio, Pisa, Italy; Cardiothoracovascular Department, Division of Cardiology, Azienda Sanitaria Universitaria Giuliano Isontina and University of Trieste, Trieste, Italy; Cardiothoracovascular Department, Division of Cardiology, Azienda Sanitaria Universitaria Giuliano Isontina and University of Trieste, Trieste, Italy; Fondazione Toscana G. Monasterio, Pisa, Italy; Department of Surgical, Medical and Molecular Pathology and of Critical Area, University of Pisa, Via Savi 10, 56126 Pisa, Italy; Department of Surgical, Medical and Molecular Pathology and of Critical Area, University of Pisa, Via Savi 10, 56126 Pisa, Italy; Department of Surgical, Medical and Molecular Pathology and of Critical Area, University of Pisa, Via Savi 10, 56126 Pisa, Italy; Department of Surgical, Medical and Molecular Pathology and of Critical Area, University of Pisa, Via Savi 10, 56126 Pisa, Italy; Cardiothoracovascular Department, Division of Cardiology, Azienda Sanitaria Universitaria Giuliano Isontina and University of Trieste, Trieste, Italy; Fondazione Toscana G. Monasterio, Pisa, Italy; Department of Clinical and Experimental Medicine, University of Messina, Messina, Italy

**Keywords:** late gadolinium enhancement, myocardial infarction, hypertrophic cardiomyopathy, prognosis

## Abstract

**Aims:**

Late gadolinium enhancement (LGE) quantification by cardiovascular magnetic resonance is central to risk stratification in hypertrophic cardiomyopathy (HCM). Conventional techniques require contour tracing and region of interest (ROI) placement, which may reduce reproducibility and increase analysis time. We developed a novel visual approach, the visual standardized quantification (VISTAQ) of LGE, that does not require myocardial contouring, arbitrary ROI positioning, or dedicated post-processing software.

**Methods and results:**

LGE images from 400 patients (100 prior myocardial infarction, 250 HCM, and 50 other non-ischaemic heart diseases) were analysed. Reproducibility was assessed using the intra-class correlation coefficients (ICC) and Bland–Altman analysis. VISTAQ was compared with conventional methods [mean+2SD, +5SD, +6SD, full-width-at-half-maximum (FWHM), visual thresholding]. Prognostic performance was evaluated in 250 HCM patients. VISTAQ demonstrated excellent intra- and inter-observer reproducibility (ICC up to 0.98 and 0.97, respectively), consistent across disease subtypes. VISTAQ showed similar ICC to FWHM but significantly lower net and absolute inter-observer differences (median absolute difference: 1.3%). Mean+2SD markedly overestimated LGE, whereas mean+6SD slightly underestimated LGE compared with VISTAQ, mean+5SD, FWHM, and visual thresholding. Analysis time was shorter with VISTAQ (median 105 vs. 375 s, *P* < 0.0001). During follow-up, 21 hard cardiac events occurred in HCM population. An LGE threshold >10% predicted events with higher accuracy using VISTAQ [area under the curve (AUC): 0.90; sensitivity: 85%; specificity: 94%) compared with mean+6SD (AUC: 0.75; sensitivity: 57%; specificity: 93%).

**Conclusion:**

VISTAQ provides highly reproducible, time-efficient LGE quantification without dedicated software and demonstrates non-inferior prognostic discrimination in HCM compared with conventional threshold-based techniques.

## Introduction

Late gadolinium enhancement (LGE) imaging is one of the cornerstone techniques in cardiovascular magnetic resonance (CMR). It enables robust tissue characterization and allows differentiation between ischaemic myocardial injury, typically following coronary artery disease, and non-ischaemic patterns of damage as seen in myocarditis and various cardiomyopathies.^1^ Beyond its diagnostic value, LGE burden has a well-established prognostic impact across a broad spectrum of cardiac diseases. In hypertrophic cardiomyopathy (HCM), in particular, quantitative assessment of LGE is essential, as current European Society of Cardiology guidelines recognize an extent ≥15% of left ventricular (LV) mass as a marker associated with adverse outcomes.^2^

Despite this central role, the optimal method for LGE quantification remains debated. Current guidelines focus primarily on LGE extent thresholds for risk stratification rather than prescribing a specific quantification technique, although a signal intensity (SI) threshold defined as the mean+2SD of a region of interest (ROI) placed in visually normal myocardium has been widely adopted in clinical practice.^2^ However, multiple studies have demonstrated that this approach is inaccurate.^3–5^ In inversion-recovery LGE sequences, normal myocardium is effectively nulled, and its residual signal mainly represents background noise, which does not follow a Gaussian distribution as typically assumed for parameters of human populations.^3^ Alternative methods, such as a threshold of mean+6SD, lack a strong physiological rationale but tend to match more closely the visual assessment of experienced readers. The full-width-at-half-maximum (FWHM) technique is another commonly used approach, yet it is limited by the need to place an ROI within areas of enhancement and performs suboptimally in the presence of small, heterogeneous, or patchy scars.^4^ All these techniques require manual delineation of endocardial and epicardial borders and the placement of ROIs in presumed normal (or enhanced) myocardium, steps that substantially increase intra-observer variability and may undermine the reproducibility of LGE quantification.

In this study, we proposed a novel LGE quantification approach, the visual standardized quantification (VISTAQ) of LGE, based on a structured visual assessment that did not require manual contouring of the myocardium nor arbitrary placement of ROIs in either remote or enhanced regions and did not require post-processing software. Specifically, we (i) compared the performance of VISTAQ against conventional methods for LGE quantification, (ii) assessed the intra- and inter-observer reproducibility of VISTAQ, and (iii) evaluated and compared the prognostic value of VISTAQ and the mean+6SD technique in a cohort of patients with HCM.

## Methods

In this multicentre, multivendor study, we retrospectively analysed LGE images from patients with prior myocardial infarction (occurred >6 months before CMR), patients with HCM, and patients with other causes of non-ischaemic heart disease.

Data of patients’ enrolment for the reproducibility study and for prognostic study are reported in the flow diagram of Supplementary data online, *Figures S1* and *S2*.

HCM was defined as a maximal LV wall thickness ≥15 mm in one or more LV myocardial segments in the absence of secondary causes of LV hypertrophy, with the exception of patients with systemic hypertension under effective pharmacological treatment who also had a family history of HCM or a consistent pathogenic genotype.^2^ Patients with suboptimal LGE image quality were excluded.

### CMR acquisition protocol

CMR examinations were performed on 1.5-T scanners from different vendors (Signa HDx, GE Healthcare, Milwaukee, WI, USA; Magnetom Avanto, Siemens Medical Systems, Erlangen, Germany; Gyroscan NT, Philips Healthcare, Amsterdam, The Netherlands) using dedicated cardiac coils. The study protocol included functional assessment with conventional short-axis cine images acquired from the mitral valve plane to the LV apex using a steady-state free precession pulse sequence. Additional cine images with identical parameters were acquired in standard two-, three-, and four-chamber views.

LGE images were acquired 10 min after administration of a gadolinium-based contrast agent at a dose of 0.2 mmol/kg in contiguous short-axis slices. An inversion-recovery T1-weighted fast gradient-echo sequence was used with the following typical parameters: field of view: 350–400 mm, slice thickness: 8 mm, 0-mm interslice gap, repetition time: 3–5 ms, echo time: 1–3 ms, flip angle: 18–25°, acquisition matrix: 224 × 224, and reconstruction matrix: 256 × 256. The inversion time was individually adjusted to null normal myocardium using a TI-scout sequence. The use of a phase-sensitive inversion-recovery (PSIR) technique was left to the discretion of the operators at each participating centre.

### Quantification of LGE extent by the conventional methods

In each centre, LGE images were analysed by experienced readers with level III CMR accreditation from the European Association of Cardiovascular Imaging, who were blinded to all clinical data. LGE extent was quantified using a commercially available research software package (CVI42, Circle Imaging).

Endocardial and epicardial contours were manually traced on each short-axis slice to delineate the LV myocardium. LGE extent was quantified using five different methods: (i) mean+2SD, (ii) mean+5SD, (iii) mean+6SD, (iv) the FWHM method, and (v) a manual threshold method.

For the mean+2SD, +5SD, and +6SD methods, a ROI was placed in visually normal remote myocardium without detectable LGE to determine the mean SI and its SD. Myocardial voxels with SI values >2SD, >5SD, and >6SD above the mean of the reference region were classified as enhanced, respectively.

For the FWHM method, an additional ROI was placed in a visually identified region of enhanced myocardium, and the SI threshold for LGE was defined according to previously published methodology.

In the manual threshold method, the SI threshold was manually selected by the investigators by progressively increasing the threshold value using a slider tool and comparing the resulting parametric maps with the original LGE images. The final threshold was defined as the value that most closely matched the visual interpretation of LGE.

The percentage of enhanced voxels within the entire LV myocardium was subsequently calculated. LGE extent was expressed both in grams and as a percentage of total LV mass.

**Figure 1:**
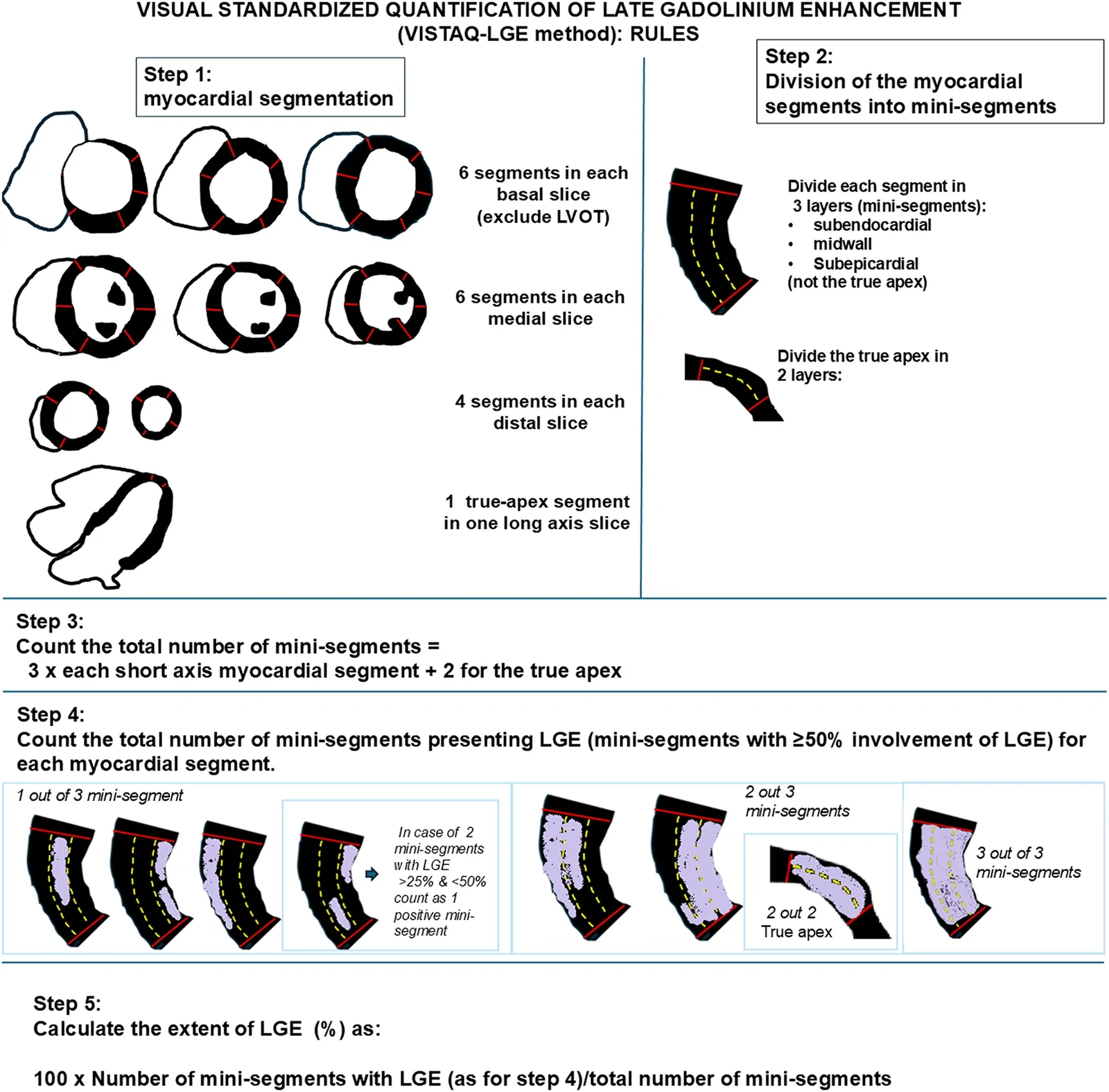
Rules of VISTAQ analysis for quantification of LGE extent. Schematic representation of the VISTAQ method for LGE quantification. Step 1: Myocardial segmentation is performed on all the short-axis slices, dividing basal and mid-ventricular levels into six segments each (excluding the LVOT), distal slices into four segments, while true apex was considered as one segment. Step 2: Each myocardial segment is further subdivided into three mini-segments (endocardial, mid-wall, and subepicardial), while the true apex is divided into mini-segments. Step 3: For each slice, the total number of mini-segments is counted (3 × each short-axis myocardial segment + 2 for the true apex). Step 4: The number of mini-segments showing LGE involvement ≥50% is visually assessed and recorded. Step 5: The extent of LGE is calculated as the percentage of positive mini-segments relative to the total number of evaluable mini-segments.

VISTAQ-LGE was assessed using the novel method called VISTAQ. The VISTAQ methodology is summarized in *Figure 1*. Briefly, for each short-axis slice, the LV myocardium was segmented as follows: six segments in each basal slice, six segments in each mid-ventricular slice, and four segments in each distal slice. In addition, a single ‘true apex’ segment was defined on a long-axis view.

In a second step, each short-axis myocardial segment was subdivided into three concentric transmural layers (‘mini-segments’: subendocardial, mid-wall, and subepicardial), whereas the true apex segment was subdivided into two layers. The total number of mini-segments was therefore calculated as: 3 × (number of short-axis segments) + 2 (true apex). LGE was then visually scored in each mini-segment, which was classified as LGE-positive when ≥50% of its area showed hyper-enhancement. When two or more mini-segments within the same segment visually exhibited an LGE extent <50% but approximately involving one-third of each mini-segment, they were collectively counted as one positive mini-segment.

For each myocardial segment, the number of LGE-positive mini-segments (0, 1, 2, or 3 in short-axis segments; 0, 1, or 2 in the true apex) was recorded. The global extent of LGE was finally expressed as a percentage of myocardial involvement, computed as:


$$\mathrm{LGE}\;\mathrm{extent}\;(\text{\%})=100\;\times \;\frac{\mathrm{number}\;\mathrm{of}\;\mathrm{positive}\;\mathrm{LGE}\;\mathrm{mini}\text{-}\mathrm{segment}}{\mathrm{total}\;\mathrm{number}\;\mathrm{of}\;\mathrm{mini}\text{-}\mathrm{segments}}$$

Myocardial segmentation followed the standard approach based on short-axis slice position: basal slices were identified as those including the mitral valve plane or showing a complete myocardial ring with visible papillary muscles at their base; mid-ventricular slices were defined as those at the level of the papillary muscle bodies, and apical (distal) slices were those located beyond the papillary muscle tips. Slices containing the LV outflow tract (LVOT) were managed by excluding the segments corresponding to the LVOT region from the analysis, as these segments cannot be reliably assessed for LGE due to partial volume effects with the aortic root and the membranous septum.

Regarding the application of the 50% mini-segment involvement rule, the operator was instructed to consider whether hyper-enhancement occupied at least half of the mini-segment area. In borderline cases, the operator used side-by-side comparison of the LGE image with a mental reference of the expected mini-segment boundaries. The complementary rule, whereby two or more mini-segments within the same segment showing approximately one-third involvement each were collectively counted as one positive mini-segment, was designed to avoid systematic underestimation of LGE in cases of diffuse, low-grade enhancement spanning multiple layers.

In cases of extremely thin myocardium, as may occur in dilated cardiomyopathy, the three-layer subdivision was maintained for consistency, although the distinction between subendocardial, mid-wall, and subepicardial layers may become less precise. In these cases, the presence or absence of hyper-enhancement was evaluated on the basis of the overall signal within the mini-segment, acknowledging that transmural differentiation is inherently limited when the wall is very thin.

All CMR readers involved in VISTAQ analysis had received specific training on the method prior to the study. Training consisted of a structured tutorial session including detailed explanation of the segmentation rules, the mini-segment scoring system, and the handling of borderline cases, followed by supervised analysis of a set of representative cases. VISTAQ analyses were performed independently and blinded to the results of conventional LGE quantification methods.

Provided as Supplementary material is the file *VISTAQ LGE.html*, which was used to calculate LGE extent using the VISTAQ method. In addition, VISTAQ can be accessed online at https://www.cmr-report.com/VISTAQ (fast link using the QR code of figure 3).

For both conventional methods and VISTAQ, analysis time was measured from the moment the operator began the assessment of LGE images. For conventional methods, the recorded time included the complete workflow from manual tracing of endocardial and epicardial contours on each short-axis slice, placement of ROI in reference myocardium (and, for the FWHM method, in the area of maximal enhancement), application of the SI threshold, and review of the resulting parametric maps. The time for launching analysis software and uploading images was not included. For VISTAQ, the recorded time encompassed visual segmentation of all short-axis slices, scoring of each mini-segment, and calculation of the final LGE percentage. All operators involved in the time comparison were already familiar with the VISTAQ method, having completed the training protocol described above.

### Prognostic evaluation

A structured clinical questionnaire was completed by a physician during scheduled outpatient visits at each participating hospital, by telephone contact with patients’ relatives, or through their general practitioners. The questionnaire recorded the occurrence of hard cardiac events, defined as cardiac death, resuscitated cardiac arrest, appropriate implantable cardioverter-defibrillator (ICD) shock, antitachycardia pacing, and sustained ventricular tachycardia documented on Holter electrocardiographic (ECG) monitoring, as well as secondary end points (heart failure hospitalization, stroke, atrial fibrillation, and acute coronary syndrome).

Device interrogations were systematically reviewed by the referring cardiologist to confirm the appropriateness of ICD therapies. The occurrence of hard cardiac events was adjudicated by a panel of expert investigators.

### Statistical analysis

Values are presented as the mean ± SD or as the median [inter-quartile range (IQR)] for variables with normal and non-normal distributions, respectively. Normal distribution was assessed using the Kolmogorov–Smirnov test.

Intra- and inter-observer variability for LGE quantification with VISTAQ-LGE was assessed by calculating the intra-class correlation coefficient (ICC). The ICC was estimated using a two-way random-effects model with absolute agreement, and both single-measure and average-measure values are reported. Bland–Altman plots were generated to evaluate intra- and inter-observer agreement across the different groups of readers. Reproducibility was classified as ‘poor’ for ICC values <0.50, ‘moderate’ for values between 0.50 and 0.75, ‘good’ for values between 0.75 and 0.90, and ‘excellent’ for values >0.90.

A receiver operating characteristic (ROC) curve was used to identify the optimal LGE threshold, measured with VISTAQ-LGE method, for predicting cardiac events. Kaplan–Meier time-to-event analysis was performed to estimate and compare event-free survival across groups. Cox regression univariate analysis was used to assess the impact of variables that were significant in on the occurrence of hard cardiac end points. A *P* < 0.05 was considered statistically significant.

## Results

### Intra- and inter-observer reproducibility

The evaluation of intra- and inter-observer reproducibility of VISTAQ was performed in a population of 250 patients (100 with previous myocardial infarction, 100 with HCM, and 50 other non-ischaemic cardiac diseases). Characteristics of this population are summarized in *Tables 1–3*.

**Table 1:** Characteristics of the population with previous myocardial infarction

Variables		Value
*n*		100
Weight, kg	Median (25th–75th)	80 (67–100)
Height, cm	Median (25th–75th)	170 (156–175)
Age	Mean ± SD	52 ± 12
Males	*n* (%)	83 (83%)
Systemic hypertension	*n* (%)	55 (55%)
Hypercholesterolemia	*n* (%)	56 (56%)
Diabetes	*n* (%)	18 (18%)
Smoking	*n* (%)	61 (61%)
NYHA > 1	*n* (%)	24 (24%)
Coronary vessel disease		
Multi vessel CAD (>1)	*n* (%)	38 (38%)
Single vessel CAD	*n* (%)	62 (62%)
LAD involvement	*n* (%)	66 (66%)
RCA involvement	*n* (%)	49 (49%)
LCx involvement	*n* (%)	33 (33%)
Left main involvement	*n* (%)	2 (2%)
Therapy		
Beta blockers	*n* (%)	50 (50%)
Calcium antagonists	*n* (%)	2.5 (2.5%)
Antiarrhythmics (amiodarone)	*n* (%)	2.5 (2.5%)
Diuretics	*n* (%)	17 (17%)
ACE inhibitors/ARB	*n* (%)	51 (51%)
Aldosterone blockers	*n* (%)	12 (12%)
CMR findings		
LV EDVi (mL/m^2^)	Mean ± SD	89 ± 22
LV ESVi (mL/m^2^)	Mean ± SD	43 ± 18
LV EF (%)	Mean ± SD	53 ± 11
LVMi (g/m^2^)	Mean ± SD	76 ± 19
WMSI	Median (25th–75th)	1.4 (1.18–1.82)
RV EDVi (mL/m^2^)	Mean ± SD	69 ± 18
RV ESVi (mL/m^2^)	Mean ± SD	24 ± 10
RV EF (%)	Mean ± SD	65 ± 11
LGE (% of LV mass)	Median (25th–75th)	12 (6–19)

ACE, angiotensin converting enzyme; CAD, coronary artery disease; EDVi, end-diastolic volume index; EF, ejection fraction; ESVi, end-systolic volume index; LAD, left anterior descending artery; LCx, left circumflex; LGE, late gadolinium enhancement; LV, left ventricle; RCA, right coronary artery; RV, right ventricle; WMSI wall motion score index.

**Table 2:** Clinical characteristics of the population of HCM for reproducibility of VISTAQ

Variables		Value
*n*		100
Age (years)	Mean ± SD	52 ± 15
Males	*n* (%)	71
Systemic hypertension	*n* (%)	7
Hypercholesterolemia	*n* (%)	16
Diabetes	*n* (%)	8
Smoking	*n* (%)	10
Dyspnoea (NYHA class > I)	*n* (%)	35
5-year HCM risk SCD < 4%	*n* (%)	85
5-year HCM risk SCD 4–6%	*n* (%)	15
Therapy		
Beta blockers	*n* (%)	46
Calcium antagonists	*n* (%)	8
Antiarrhythmics	*n* (%)	3
Diuretics	*n* (%)	12
ACE inhibitors	*n* (%)	33
Aldosterone blockers	*n* (%)	3
CMR findings:		
Maximum wall thickness ≥30 mm	*n* (%)	4
Family history of SCD	*n* (%)	19
Syncope	*n* (%)	9
Non-sustained VT	*n* (%)	44
Obstructive HCM	*n* (%)	20
LV outflow gradient (mmHg)	Mean ± SD	53 ± 30
Left atrial diameter (mm)	Median (25th–75th)	32 (30–43)
LV EDVi (mL/m^2^)	Mean ± SD	69 ± 17
LV ESVi (mL/m^2^)	Mean ± SD	21 ± 8
LV EF (%)	Mean ± SD	68 ± 12
LVMi (g/m^2^)	Mean ± SD	114 ± 51
RV EDVi (mL/m^2^)	Mean ± SD	63 ± 17
RV ESVi (mL/m^2^)	Mean ± SD	20 ± 8
RV EF (%)	Mean ± SD	69 ± 9
LGE (%)	Median (25th–75th)	2 (1–9)

ACE, angiotensin converting enzyme; EDVi, end-diastolic volume index; EF, ejection fraction, RV, right ventricle; ESVi, end-systolic volume index; HCM, hypertrophic cardiomyopathy; LGE, late gadolinium enhancement; LV, left ventricle; SCD, sudden cardiac death; VT, ventricular tachycardia.

**Table 3:** Characteristics of the non-ischaemic cardiac disease population

Characteristic	
*n*	50
Males, *n* (%)	39 (78%)
Age, years	42 (22–55)
Weight, kg	73 ± 15
Height, cm	171 ± 10
Type of NICD	
Previous myocarditis	30 (60%)
DCM	10 (20%)
NDLVC	10 (20%)
Risk factors	
Hypertension	1 (2%)
Hypercholesterolemia	1 (2%)
Diabetes mellitus	1 (2%)
Smoking	2 (4%)
Family history of CAD	3 (6%)
CMR data	
LVEDVi, mL/m^2^	89 (68–98)
LVESVi, mL/m^2^	30 (25–38)
LV EF, %	55 (44–66)
WMSI >1, *n*	10 (21%)
LVMi, g/m^2^	68 (62–70)
RVEDVi, mL/m^2^	80 (66–92)
RVESVi, mL/m^2^	32 (26–39)
RV EF, %	59 (54–69)
LGE extent, % of LV mass	2 (0–5)

CAD, coronary artery disease; CMR, cardiac magnetic resonance; DCM, dilated cardiomyopathy; EF, ejection fraction; LGE, late gadolinium enhancement; LV, left ventricle; LVEDVi, left ventricular end-diastolic volume index; LVESVi, left ventricular end-systolic volume index; LVMi, left ventricular mass index; NDLVC, non-dilated left ventricular cardiomyopathy; NICD, non-ischaemic cardiac disease; RV, right ventricle; RVEDVi, right ventricular end-diastolic volume index; RVESVi, right ventricular end-systolic volume index; WMSI, wall motion score index.

In the whole population, quantification of LGE extent by VISTAQ method demonstrated an excellent intra-observer reproducibility [single measures ICC 0.96, 95% confidence interval (CI) 0.95–0.97, average measures ICC 0.98, 95% CI 0.97–0.99], an excellent inter-observer reproducibility (single measures ICC 0.94, 95% CI 0.91–0.95, average measures ICC 0.97, 95% CI 0.96–0.97).

**Figure 2:**
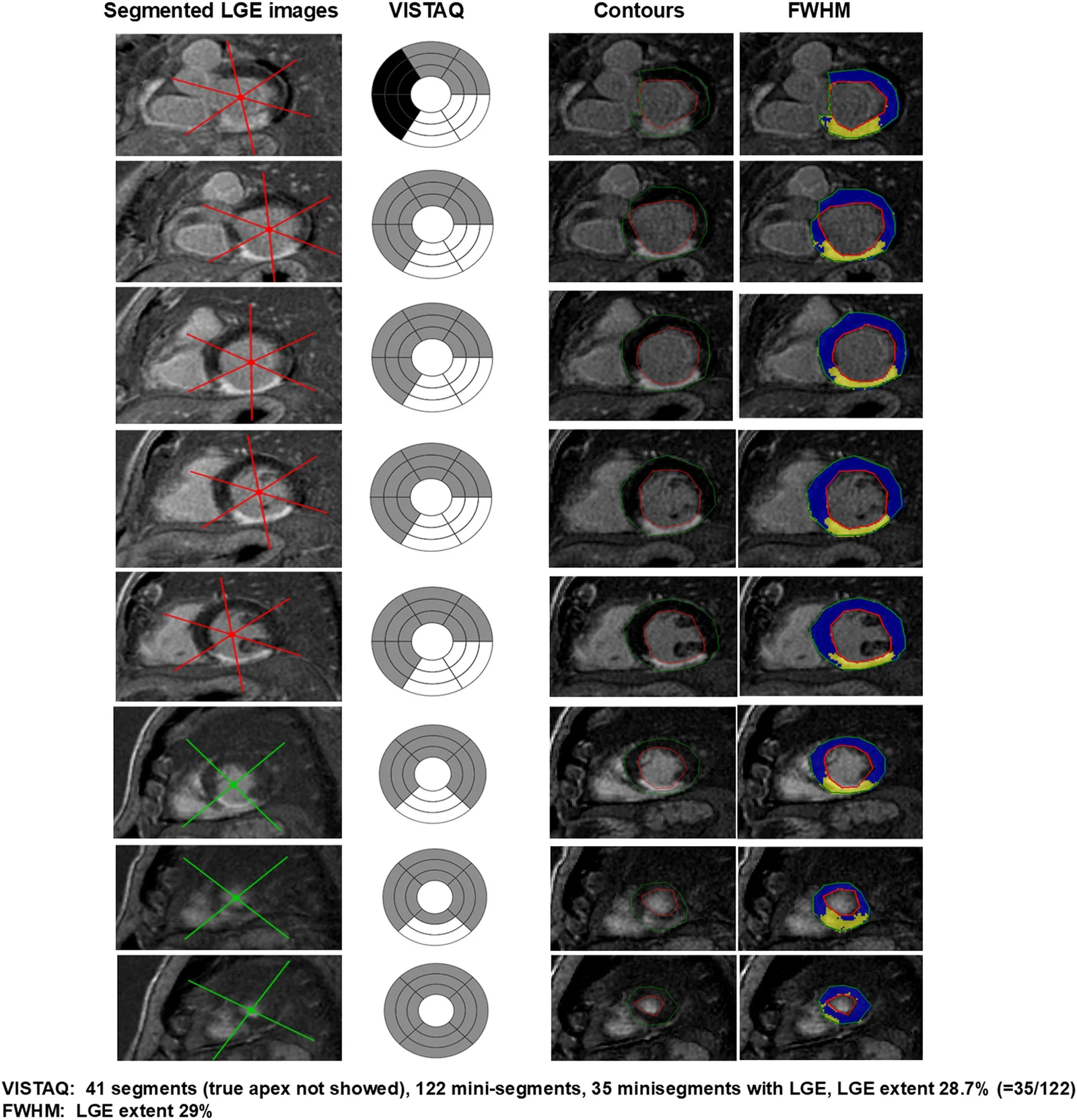
Example of VISTAQ analysis in a patient with previous myocardial infarction. In the first column, original LGE images are shown with myocardial segmentation into six segments (basal and mid-ventricular slices) or four segments (distal slices). In the second column, VISTAQ analysis was performed on bull’s-eye plots generated from this segmentation. Black indicates mini-segments excluded from analysis because they are located at the level of the LVOT, grey indicates mini-segments without LGE, and white indicates mini-segments with LGE. In this case, a total of 122 mini-segments were analysed, of which 35 showed LGE, corresponding to an LGE extent of 28.7% of LV mass. In the third column, endocardial and epicardial contours of the same patient’s LGE images are shown, while the fourth column displays the parametric map obtained using the FWHM method. With this approach, LGE extent was 28.8%.

In the population of patients with previous myocardial infarction, VISTAQ demonstrated an excellent intra-observer reproducibility (single measures ICC 0.94, 95% CI 0.90–0.97, average measures ICC 0.97, 95% CI 0.95–0.98), and a very good/excellent inter-observer reproducibility (single measures ICC 0.91, 95% CI 0.87–0.93, average measures ICC 0.94, 95% CI 0.90–0.96). Example of VISTAQ analysis compared with FWHM in a case of previous myocardial infarction is shown in *Figure 2*.

**Figure 3:**
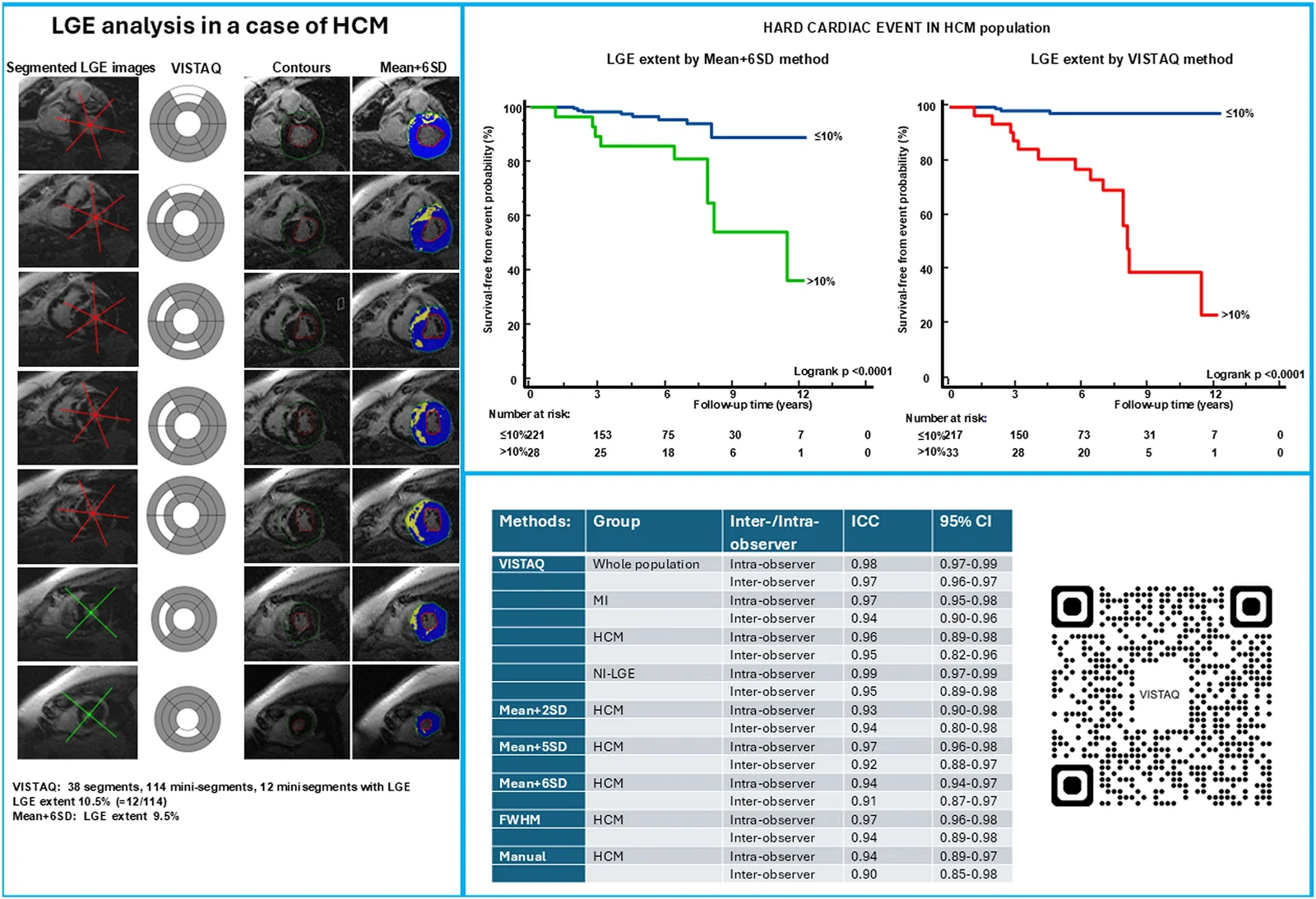
The VISTAQ-LGE () method is a novel method to estimate the extent of LGE without the need for post-processing software, contour tracing nor placement of ROI. In the left panel, it is shown an example of quantification of LGE using mean+6SD and VISTAQ in a patient with HCM. In the right upper panel, Kaplan–Meier survival-free from hard cardiac events curves in patients with HCM are shown. Patients with LGE extent >10%, measured both with the mean+6SD method and with VISTAQ, had worse prognosis than those with lower LGE extent In the lower right panel, a table summarizes the ICC of reproducibility for LGE extent measurement by VISTAQ and other conventional methods (mean+2SD, +5SD, +6SD, FWHM, and visual threshold). In the same panel, the QR code links to an easy tool to use the VISTAQ method.

In the population of patients with HCM, VISTAQ demonstrated an excellent intra-observer reproducibility (single measures ICC 0.92, 95% CI 0.81–0.96, average measures ICC 0.96, 95% CI 0.89–0.98), and very good/excellent inter-observer reproducibility (single measures ICC 0.91, 95% CI 0.70–0.93, average measures ICC 0.92, 95% CI 0.82–0.96). Example of VISTAQ analysis compared with mean+6SD in a case of HCM is shown in *Figure 3*.

**Figure 4:**
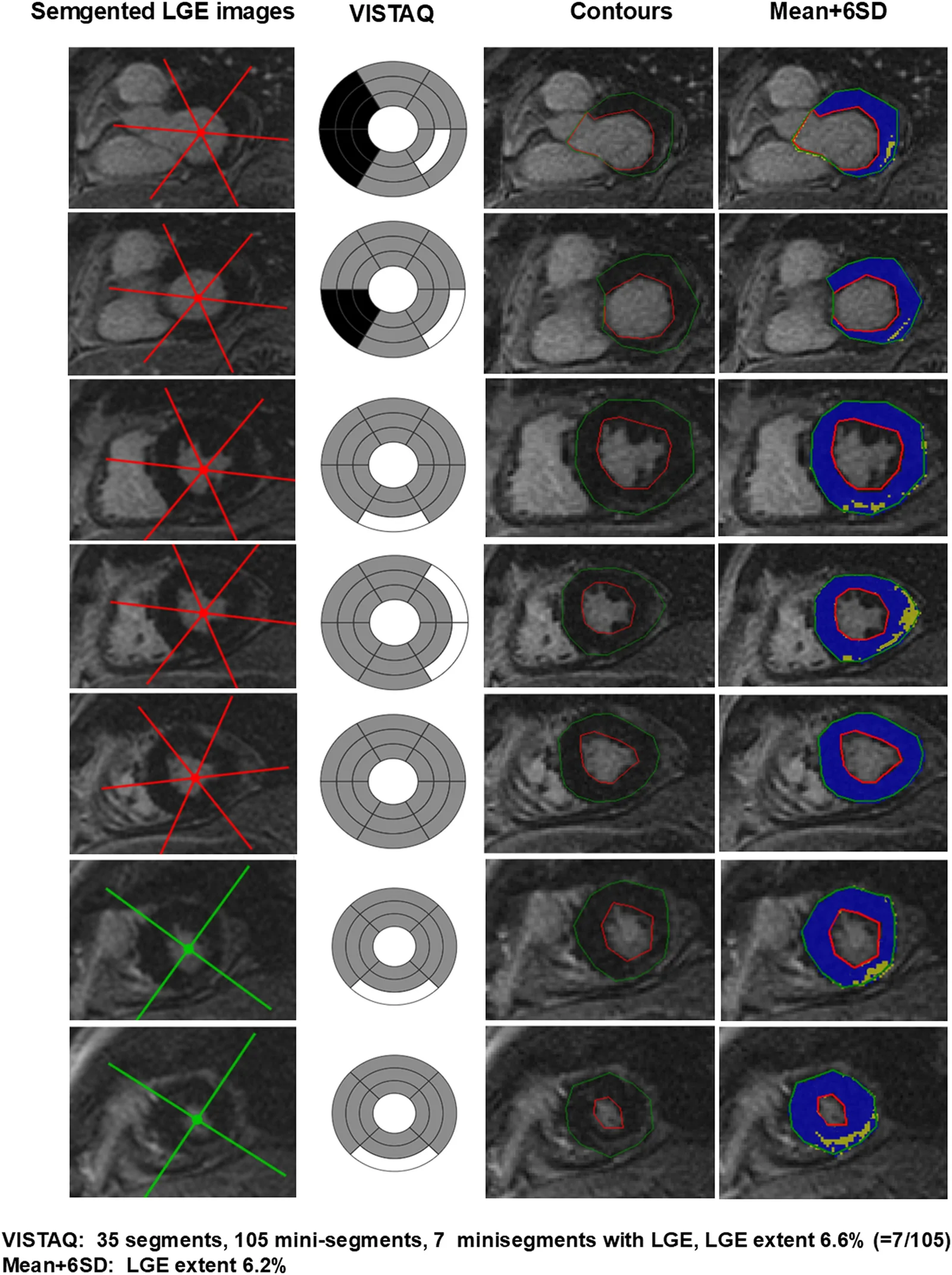
Example of quantification of LGE using mean+6SD and VISTAQ in a patient with non-ischaemic fibrosis (previous myocarditis).

In the population of patients with non-ischaemic LGE without LV hypertrophy, VISTAQ demonstrated an excellent intra-observer reproducibility (single measures ICC 0.98, 95% CI 0.94–0.99, average measures ICC 0.99, 95% CI 0.97–0.99), and a very good/excellent inter-observer reproducibility (single measures ICC 0.91, 95% CI 0.81–0.96, average measures ICC 0.95, 95% CI 0.89–0.98). Example of VISTAQ analysis compared with mean+6SD in a case of previous myocarditis is shown in *Figure 4*.

### Comparison with conventional methods

The comparison of reproducibility among VISTAQ and other conventional methods for LGE quantification (mean+2SD, +5SD, +6SD, FWHM, and manual threshold methods) were performed in 100 patients with HCM. ICCs for intra- and inter-observer reproducibility of conventional techniques are shown in *Table 4*.

**Table 4:** Intra-class correlation coefficients

Methods	Group	Inter-/intra-observer	ICC (single)	95% CI	ICC (average)	95% CI
VISTAQ	Whole population	Intra-observer	0.96	0.95–0.97	0.98	0.97–0.99
		Inter-observer	0.94	0.91–0.95	0.97	0.96–0.97
	MI	Intra-observer	0.94	0.90–0.97	0.97	0.95–0.98
		Inter-observer	0.91	0.82–0.93	0.94	0.90–0.96
	HCM	Intra-observer	0.92	0.81–0.96	0.96	0.89–0.98
		Inter-observer	0.91	0.70–0.93	0.95	0.82–0.96
	NI-LGE	Intra-observer	0.98	0.94–0.99	0.99	0.97–0.99
		Inter-observer	0.91	0.81–0.96	0.95	0.89–0.98
Mean+2SD	HCM	Intra-observer	0.90	0.88–0.95	0.93	0.90–0.98
		Inter-observer	0.79	0.67–0.95	0.94	0.80–0.98
Mean+5SD	HCM	Intra-observer	0.90	0.87–0.97	0.97	0.96–0.98
		Inter-observer	0.78	0.75–0.94	0.92	0.88–0.97
Mean+6SD	HCM	Intra-observer	0.92	0.87–0.97	0.94	0.94–0.97
		Inter-observer	0.78	0.76–0.94	0.91	0.87–0.97
FWHM	HCM	Intra-observer	0.95	0.92–0.96	0.97	0.96–0.98
		Inter-observer	0.91	0.89–0.98	0.94	0.89–0.98
Manual	HCM	Intra-observer	0.92	0.88–0.94	0.94	0.89–0.97
		Inter-observer	0.76	0.75–0.97	0.90	0.85–0.98

The median difference of LGE extent between two different investigators was calculated for each method as net difference (including negative numbers) and as absolute difference (used absolute value of negative numbers). VISTAQ method showed both lower net (median 0, IQR −1.8–0.8) and absolute (median 1.3, IQR 0.3–3.4) difference than FWHM (median of net difference 2.5, IQR 0.2–5.8, *P* < 0.0001; median of absolute difference 3.1, IQR 1.5–5.8, *P* < 0.0001), mean+2SD (median of net difference 4.8, IQR −1.3–9.8, *P* < 0.0001; median of absolute difference 5.2, IQR 2.6–9.6, *P* < 0.0001), mean+5SD (median of net difference −2.2, IQR −4.4–0.4, *P* = 0.008; median of absolute difference 2.8, IQR 1.3–4.7, *P* = 0.007), mean+6SD (median of net difference −1.5, IQR −3.5–0.8, *P* = 0.04; median of absolute difference 2.1, IQR 1–3.8, *P* = 0.02) and visual threshold (median of net difference −2.7, IQR −5.9 to −0.6, *P* < 0.0001; median of absolute difference 3.5, IQR 1.8–6.5, *P* < 0.0001).

Overall, the mean+2SD method significantly overestimated LGE extent (median extent 22%, IQR 15–33) compared with mean+5SD (median extent 8%, IQR 4–15, *P* < 0.0001), to mean+6SD (median extent 7%, IQR 2–12, *P* < 0.0001), to FWHM (median extent 9%, IQR 5–16, *P* < 0.0001), to visual threshold method (median extent 8%, IQR 6–10, *P* < 0.0001), and to VISTAQ (median extent 9%, IQR 5–15, *P* < 0.0001).

In contrast, LGE extent measured by the mean+6SD method was significantly lower than by mean+5SD (*P* = 0.02), by FWHM (*P* = 0.002), by visual threshold (*P* = 0.002) and by VISTAQ (*P* = 0.002).

No significant differences were found when LGE extent was measured by mean+5SD, FWHM, visual threshold, and VISTAQ.

**Figure 5:**
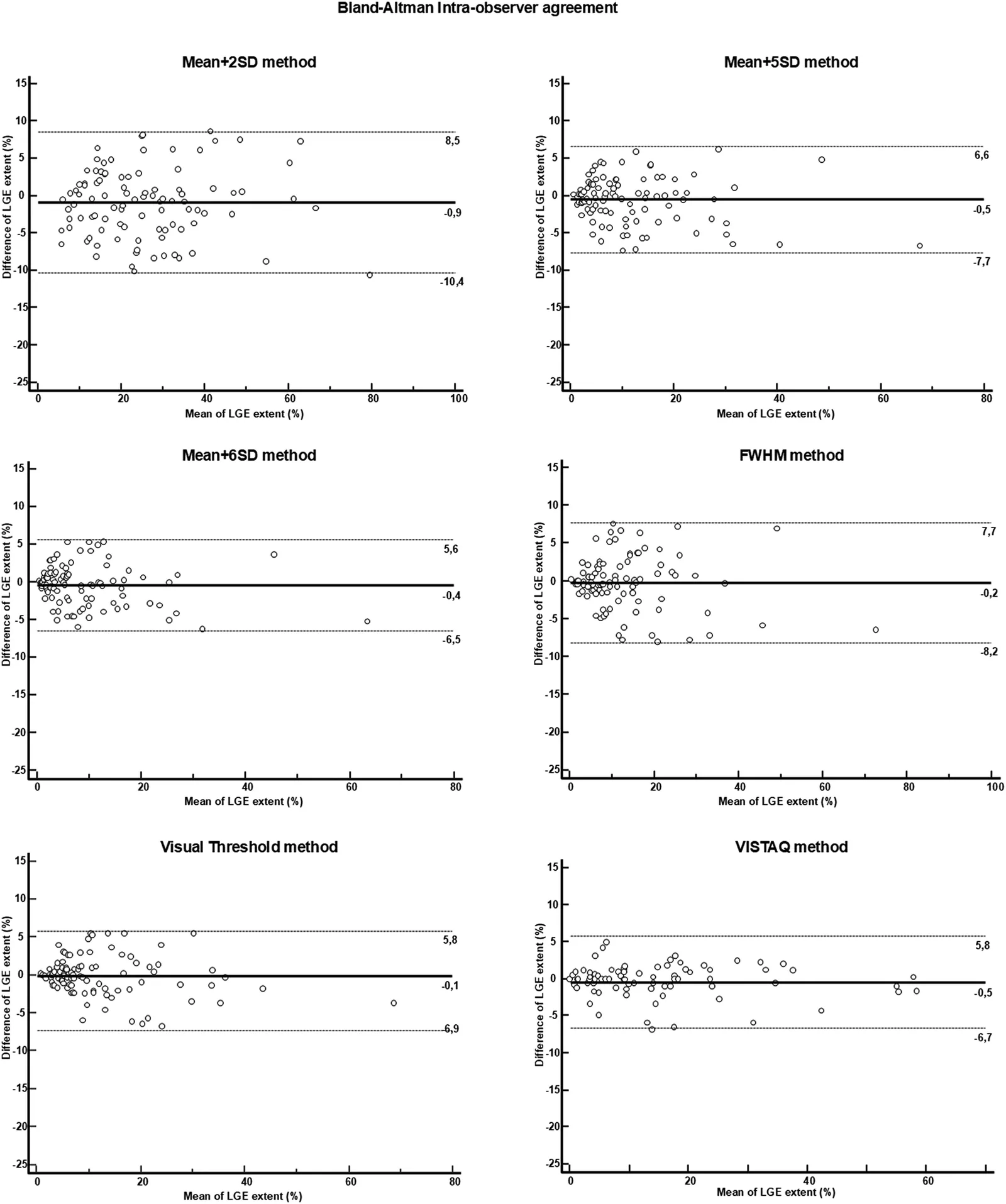
Bland–Altman plots for intra-observer agreement for quantification of LGE using different method of analysis.

**Figure 6:**
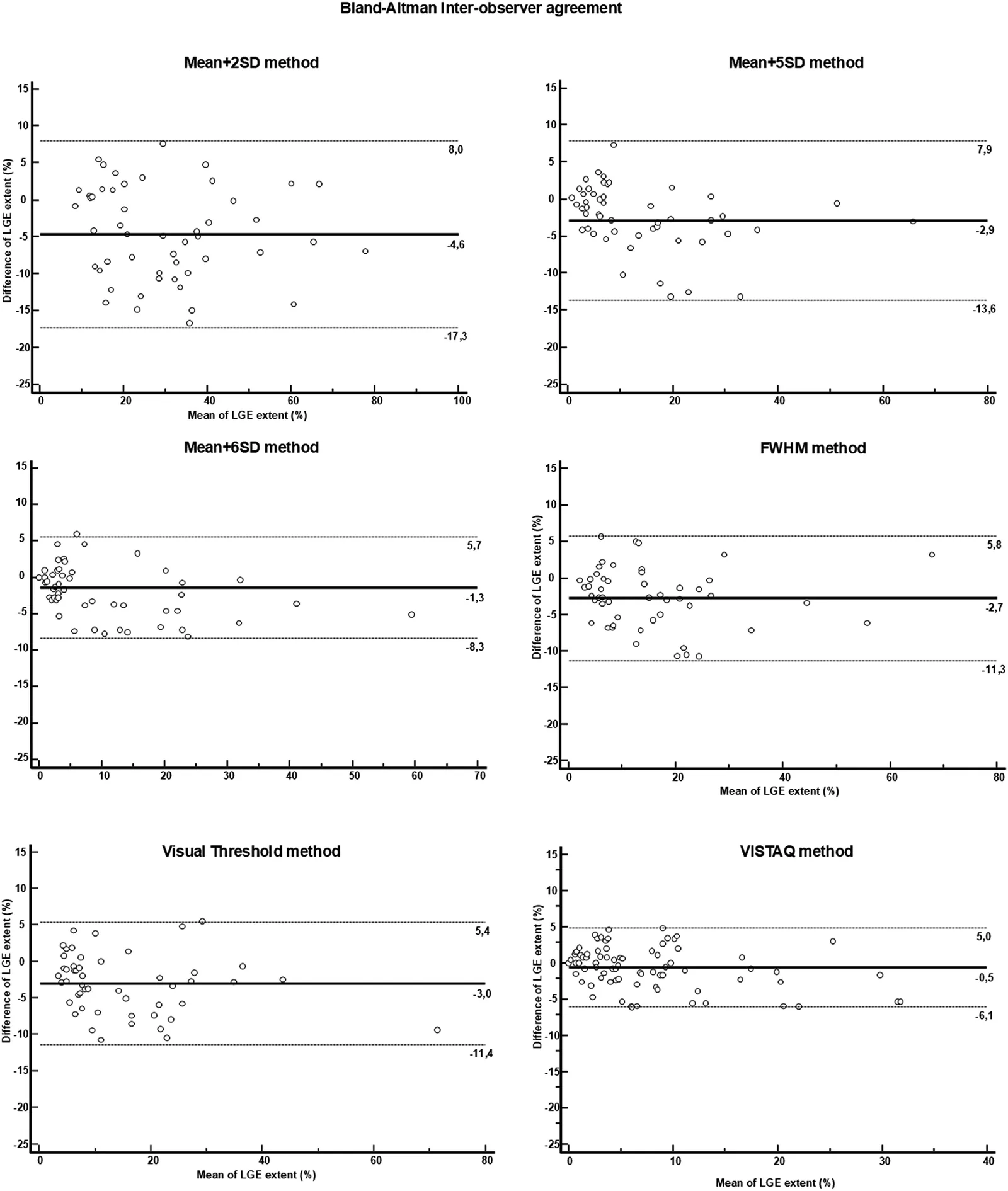
Bland–Altman plots for inter-observer agreement for quantification of LGE using different method of analysis.

Bland–Altman plots for intra- and inter-observer agreement for all the methods of LGE quantification are shown respectively in *Figures 5* and *6*. As shown in *Figure 5*, in the intra-observer analysis, the mean differences (biases) were similar across techniques; however, VISTAQ showed narrower 95% limits of agreement (LoAs): VISTAQ, bias −0.5, 95% LoAs −6.7 to 5.8; mean+2SD, bias −0.9, 95% LoAs −10.4 to 8.5; mean+5SD, bias −0.5, 95% LoAs −7.7 to 6.6; mean+6SD, bias −0.4, 95% LoAs −6.5 to 5.6; FWHM, bias −0.2, 95% LoAs −8.2 to 7.7; and the visual threshold method, bias −0.1, 95% LoAs −6.9 to 5.8.

In contrast, in the inter-observer analysis (*Figure 6*), VISTAQ showed a smaller absolute bias (−0.5; 95% LoAs, −6.1 to 5.0) than mean+2SD (−4.6; 95% LoAs, −11.3 to 8.0), mean+5SD (−2.9; 95% LoAs, −13.6 to 7.9), mean+6SD (−1.3; 95% LoAs, −8.3 to 5.7), FWHM (−2.7; 95% LoAs, −11.3 to 5.8), and the visual threshold method (−3.0; 95% LoAs, −11.4 to 5.4).

### Time-duration of analysis

The median time-duration required for the analysis of LGE with VISTAQ was significantly lower than that with mean+2SD, +5SD, +6SD, FWHM and manual threshold, a median of 105 (25–75th 70–130) vs. 375 (25–75th 277–550) s, *P* < 0.0001.

### Prognostic role of LGE extent by VISTAQ in HCM

The evaluation of prognostic role of LGE extent assessed by VISTAQ was performed in a population of 250 patients with HCM (*Table 5*). During the median follow-up of 5 years,^4–10^ hard cardiac events occurred in 21 patients (five episodes of cardiac sudden death; eight resuscitated cardiac arrests; one episode of sustained ventricular tachycardia; and seven appropriate interventions of ICD). At ROC analysis, the best cut-off of LGE extent measured by mean+6SD for predicting hard cardiac event method was >10% with area under the curve (AUC) of 0.75 (95% CI 0.69–0.82, sensitivity: 57%, and specificity: 93%). The same threshold of >10% was found for LGE extent measured by VISTAQ method but with higher AUC (0.90, 95% CI 0.85–0.93), sensitivity (85%, 95% CI 64–97%), and specificity (94%, 95% CI 90–96%).

**Table 5:** Clinical characteristics of the population of HCM for prognostic assessment of LGE extent measured by VISTAQ method

Variables		Value
*n*		250
Age (years)	Mean ± SD	54 ± 17
Males	*n* (%)	180 (72%)
Systemic hypertension	*n* (%)	18 (7%)
Hypercholesterolemia	*n* (%)	35 (14%)
Diabetes	*n* (%)	20 (8%)
Smoking	*n* (%)	25 (10%)
Dyspnoea (NYHA class > I)	*n* (%)	88 (35%)
5-year HCM risk SCD < 4%	*n* (%)	212 (85%)
5-year HCM risk SCD 4–6%	*n* (%)	38 (15%)
Therapy		
Beta blockers	*n* (%)	115 (46%)
Calcium antagonists	*n* (%)	20 (8%)
Antiarrhythmics	*n* (%)	8 (3%)
Diuretics	*n* (%)	30 (12%)
ACE inhibitors	*n* (%)	8 (3%)
Aldosterone blockers	*n* (%)	8 (3%)
CMR findings		
Maximum wall thickness ≥30 mm	*n* (%)	10 (4%)
Family history of SCD	*n* (%)	48 (19%)
Syncope	*n* (%)	23 (9%)
Non-sustained VT	*n* (%)	110 (44%)
Obstructive HCM	*n* (%)	55 (22%)
LV outflow gradient (mmHg)	Mean ± SD	59 ± 30
Left atrial diameter (mm)	Median (25th–75th)	38 (33–44)
LV EDVi (mL/m^2^)	Mean ± SD	72 ± 19
LV ESVi (mL/m^2^)	Mean ± SD	23 ± 9
LV EF (%)	Mean ± SD	69 ± 11
LVMi (g/m^2^)	Mean ± SD	114 ± 51
RV EDVi (mL/m^2^)	Mean ± SD	62 ± 17
RV ESVi (mL/m^2^)	Mean ± SD	19 ± 8
RV EF (%)	Mean ± SD	68 ± 9
LGE (%)	Median (25th–75th)	2 (1–8)
LGE > 10% of LV mass (mean+6SD)	*n* (%)	28 (11%)
LGE > 15% of LV mass (mean+6SD)	*n* (%)	15 (6%)
LGE >10% of LV mass (VISTAQ)	*n* (%)	32 (13%)
LGE >15% of LV mass (VISTAQ)	*n* (%)	22 (9%)

ACE, angiotensin converting enzyme; EDVi, end-diastolic volume index; EF, ejection fraction, RV, right ventricle; ESVi, end-systolic volume index; HCM, hypertrophic cardiomyopathy; LGE, late gadolinium enhancement; LV, left ventricle; SCD, sudden cardiac death; VT, ventricular tachycardia.

VISTAQ and mean+6SD concordantly found a LGE extent of >10% in 23 patients, but VISTAQ found LGE extent >10% in nine patients with lower extent by mean+6SD and on the contrary five patients with lower extent with VISTAQ had extent >10% using the mean+6SD technique.

The identification of patients with LGE extent >10% by VISTAQ method allowed prediction of 18 out of 21 events, whereas when measured using the mean+6SD, patients with >10% extent had 12 events. In *Figure 3*, Kaplan–Meier survival curves assessing survival-free from hard cardiac events are showed. Using both methods, an extent of LGE >10% was associated with worse prognosis (log-rank *P* < 0.0001 in both cases), but VISTAQ had greater HR than mean+6SD (HR 56, 95% CI 19–163, vs. HR 11, 95% CI 4–32, *P* < 0.0001).

## Discussion

In this study, we proposed a novel visual semi-quantitative method for quantification of LGE extent, the VISTAQ which does not require myocardial contours tracing nor ROI placement.

Results may be summarized as follows:

1. VISTAQ provides excellent intra- and inter-observer reproducibility for LGE quantification across different cardiac conditions (post-myocardial infarction, HCM, and non-ischaemic myocardial fibrosis);
2. VISTAQ had comparable reproducibility of FWHM but with lower net and absolute differences of inter-observer measurement of LGE extent;
3. VISTAQ markedly shortens analysis time compared with all conventional methods; and
4. quantification of LGE by VISTAQ retains its prognostic value in patients with HCM.

VISTAQ is actually a semi-quantitative approach based on the analysis of myocardial mini-segments; however, with standard short-axis LGE acquisitions covering the entire left ventricle, typically yielding 100–180 mini-segments, the estimation of LGE extent closely approximates a fully quantitative assessment.

A principal strength of VISTAQ is that it eliminates the need for post-processing software, myocardial contour delineation, and placement of ROI in both normal and pathological myocardium. This also translates into high reproducibility. In the overall population of 250 patients, intra-observer ICC values reached 0.96 (single measures) and 0.98 (average measures), while inter-observer ICC values were 0.94 and 0.97, respectively. These values fall within the range considered excellent and indicate minimal measurement variability attributable to observer-related factors.

Importantly, reproducibility remained consistently high across distinct pathological substrates, including previous myocardial infarction, HCM, and non-ischaemic LGE without LV hypertrophy. This finding is clinically plausible, as LGE patterns in HCM are often patchy, intramyocardial, and less sharply demarcated compared with the subendocardial or transmural patterns typical of ischaemic scar. Despite this inherent complexity, VISTAQ maintained strong consistency, underscoring its robustness even in challenging imaging phenotypes.

A possible explanation for the observed reproducibility may lie in the absence of the need to manually delineate endocardial and epicardial myocardial contours, as well as in the lack of requirement for ROI placement in either normal or pathological myocardium (as is necessary with the FWHM method).

When contouring is performed, particular care must be taken to exclude the ventricular cavity from the endocardial border. In LGE images, blood appears hyper-intense due to the presence of gadolinium-based contrast agents; inadvertent inclusion of blood pool may therefore lead to overestimation of LGE extent. To avoid this, operators often trace slightly within the endocardial border. However, this approach may reduce the calculated total myocardial mass and consequently inflate the estimated LGE burden when expressed as a percentage of ventricular mass.

Similarly, epicardial fat is hyper-intense on LGE images, and its inclusion within the epicardial contour may also result in LGE overestimation. The need for meticulous attention to these technical aspects may increase inter- and intra-observer variability when using conventional quantification methods.

ROI placement represents a critical determinant of LGE quantification in conventional approaches, including techniques based on the mean SI of remote myocardium plus 2, 5, or 6 SDs, as well as in the FWHM method, which requires ROI positioning within the area of maximal enhancement.

In conditions such as prior myocardial infarction, ROI placement in both remote myocardium and scar tissue is generally straightforward. In contrast, in non-ischaemic cardiomyopathies, particularly HCM, identification of truly unaffected myocardium may be challenging. This difficulty may also be present, although to a lesser extent, in other cardiomyopathies and myocarditis. Variability in ROI placement can lead to subtle differences in calculated mean SI and SD values, thereby altering the threshold definition and ultimately affecting final LGE quantification.

Furthermore, in cases of small, patchy, or highly irregular scars, such as those frequently observed in HCM or myocarditis, accurate ROI positioning within hyper-enhanced regions may be difficult, representing a potential limitation of the FWHM technique.

Another relevant advantage of VISTAQ is its ability to manage imaging artefacts. With conventional quantification methods, it may be challenging to exclude false hyper-intensities arising from motion artefacts, respiratory misregistration, ECG triggering issues, or suboptimal nulling of normal myocardium. These sources of error may lead to inaccurate LGE estimation when automated or semi-automated threshold-based techniques are applied.

**Figure 7:**
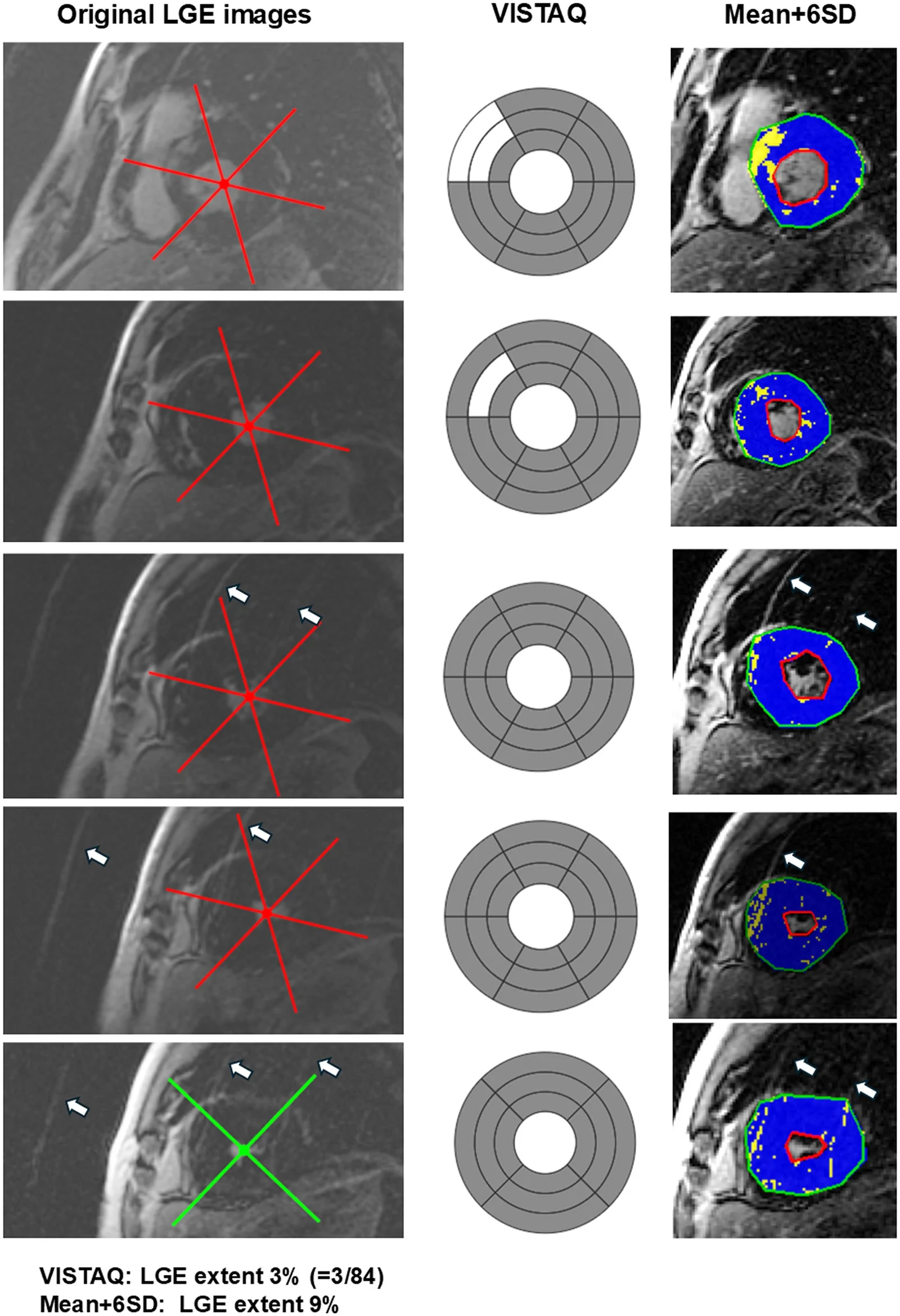
Example of VISTAQ and conventional analysis (mean+6SD method) in a patient with HCM and respiratory motion artefacts (white arrows). Operator interpretation allowed the hyper-intense respiratory motion artefacts to be recognized as non-pathological in VISTAQ method, whereas they were erroneously quantified as LGE by the conventional method. In this case, there was a mismatch between the two methods, with the conventional method clearly overestimating the extent of LGE. This case was excluded in the comparative study because of the artefacts, but it is an example of potential advantages of VISTAQ compared with conventional methods.

In contrast, VISTAQ, when used by experienced operators capable of recognizing artefact-related signal abnormalities, allows these artefactual hyper-intensities to be identified and excluded (example in *Figure 7*). This may potentially enable a more reliable estimation of LGE extent even in suboptimal image quality conditions.

The intra- and inter-observer reproducibility of conventional technique as mean+2SD, mean+5SD, mean+6SD, FWHM, and visual thresholding was evaluated in the present study in a cohort of patients with HCM. We chose to perform this comparison exclusively in HCM because, to date, LGE quantification is formally recommended only in this cardiomyopathy.^2^

Our results are consistent with previous studies demonstrating that FWHM is the most reproducible among conventional methods.^4,6^ However, VISTAQ showed intra- and inter-observer ICC values comparable to FWHM, with smaller absolute and net inter-observer differences than FWHM and all other methods.

Previous studies^6–9^ have evaluated only the net difference between observers, whereas in the present study, we assessed both the net and the absolute differences. This distinction is methodologically relevant. When considering net differences, opposite deviations may cancel each other out. For example, if in one case the difference between observer A and B is −10% and in the subsequent case it is +10%, the mean net difference would be 0%, suggesting perfect agreement. In contrast, when absolute differences are analysed, both values are treated as 10%, resulting in a mean absolute difference of 10%. Therefore, the assessment of absolute differences provides a more accurate estimate of the true magnitude of inter-observer variability, avoiding the potential masking effect inherent to net difference calculations.

The median net difference in LGE extent between observers was 0%, with narrow CIs, while the absolute median difference was only 1.3%. All comparator techniques demonstrated significantly greater disagreement, with absolute differences ranging from ∼2 to over 5%.

Additional noteworthy findings emerged from the comparison of LGE extent values obtained with the different methods when performed by the same operator. In the present study, consistent with previous evidence,^4^ the mean+2SD method proved unreliable, leading to an approximate two-fold overestimation of LGE extent.

This finding is predictable and can be explained by magnetic resonance physics. The distribution of SI in remote (healthy) myocardium on LGE images is not Gaussian. Healthy myocardium is nulled by the inversion pulse; therefore, what is measured is not true myocardial signal but background noise. Background noise does not follow a Gaussian (normal) distribution, typical of many biological variables, but rather a Rician distribution, which is not symmetric and bell-shaped like a Gaussian curve. Instead, it exhibits a broader right-sided tail towards higher values.^3^ Consequently, the mean+2SD approach, which assumes Gaussian distribution, is not appropriate for LGE quantification and results in substantial overestimation.

As the number of SDs above the mean increases, the threshold for LGE detection shifts towards higher values, inevitably reducing the estimated LGE extent.

Our results demonstrate that the mean+6SD method, by applying a higher threshold, leads to a statistically significant, albeit modest, underestimation of LGE extent compared with mean+5SD, FWHM, visual thresholding, and VISTAQ. In contrast, measurements obtained with VISTAQ, mean+5SD, FWHM, and visual thresholding did not differ significantly from one another.

The implications of this relative ‘underestimation’ are clinically relevant. In the multicentre study by Chan *et al.*,^11^ in which LGE was quantified using visual thresholding, the optimal prognostic cut-off was 15% of LV mass. Conversely, studies that quantified LGE using the 6SD method^12–14^ identified an optimal cut-off of 10% of LV mass. This discrepancy may, at least in part, be explained by the higher threshold and consequent underestimation of LGE extent inherent to the mean+6SD method compared with other techniques.

Lower variability of VISTAQ has important clinical implications. In risk stratification, particularly when clinical decisions may hinge on a fixed threshold of LGE extent, even small measurement differences can shift patients across risk categories. A more consistent technique therefore enhances the reliability of clinical decision-making.

Perhaps the most clinically meaningful finding of this study is the improved prognostic performance of VISTAQ in patients with HCM. Using a threshold of >10% LGE extent, VISTAQ achieved an AUC of 0.90, compared with 0.75 for the mean+6SD method. This difference represents a substantial gain in discriminative ability.

Notably, sensitivity increased from 57% (mean+6SD) to 85% (VISTAQ), while specificity remained similarly high (94% vs. 93%). Thus, VISTAQ identified significantly more patients who subsequently experienced hard cardiac events without increasing false positives. In absolute terms, VISTAQ correctly identified 18 of 21 events, whereas mean+6SD identified only 12.

The survival analysis further supports the clinical relevance of these findings. Although both methods showed significant prognostic separation at the >10% threshold, the hazard ratio (HR) associated with VISTAQ (HR 56) was markedly higher than that observed with mean+6SD (HR 11). While wide CIs reflect the limited number of events, the magnitude of effect suggests that VISTAQ captures a biologically meaningful representation of arrhythmogenic substrate.

From a pathophysiological standpoint, myocardial fibrosis in HCM creates conduction heterogeneity and re-entry circuits, providing the substrate for malignant ventricular arrhythmias. A quantification method that more accurately and consistently captures total fibrotic burden is therefore expected to correlate more strongly with arrhythmic outcomes. The superior performance of VISTAQ may reflect a more faithful representation of this structural substrate, avoiding both overestimation from low thresholds and underestimation from excessively stringent criteria.

Some methodological considerations related to spatial resolution and specific clinical scenarios deserve discussion. VISTAQ, as a semi-quantitative approach based on visual assessment of predefined mini-segments, inherently involves a trade-off between simplicity and spatial precision. Compared with voxel-based quantification methods, VISTAQ may offer reduced spatial resolution, particularly for very small focal scars that do not reach the 50% involvement threshold within any individual mini-segment. Similarly, insertion-point fibrosis, which is frequently observed at the right ventricular insertion points in HCM and is often limited in extent, may be underestimated by VISTAQ if the enhancement does not meet the threshold for mini-segment positivity. Conversely, in cases of diffuse patchy fibrosis, as is commonly encountered in HCM, the structured segmentation of VISTAQ may actually provide a more reproducible assessment than conventional methods, which are often challenged by the difficulty of ROI placement in heterogeneous enhancement patterns.

Regarding the applicability of VISTAQ across different imaging techniques, the method was developed and validated primarily using magnitude inversion-recovery LGE sequences. However, it is expected to be equally applicable to PSIR imaging, as VISTAQ relies on visual assessment of hyper-enhancement relative to surrounding myocardium rather than on absolute SI values. PSIR sequences may actually facilitate VISTAQ analysis by providing more uniform background suppression and improved contrast between enhanced and normal myocardium, thereby reducing ambiguity in mini-segment scoring. Similarly, VISTAQ should be compatible with high-resolution 3T imaging, which offers improved signal-to-noise ratio and spatial resolution compared with 1.5T, potentially enhancing the visualization of small areas of enhancement and improving the accuracy of mini-segment classification. Dark-blood LGE sequences, which have been increasingly adopted in modern clinical practice,^10^ represent another context in which VISTAQ could be applied. By suppressing the blood pool signal, dark-blood techniques improve the delineation of subendocardial enhancement, which may in turn facilitate more accurate visual assessment of mini-segment involvement, particularly in the subendocardial layer.

An important consideration for the clinical translation of VISTAQ is the level of training required. Although VISTAQ is designed to minimize operator dependency by eliminating contour tracing and ROI placement, visual assessment still requires familiarity with LGE patterns and the ability to distinguish true enhancement from artefacts. In the present study, all readers had level III CMR accreditation and underwent specific VISTAQ training. The performance of VISTAQ among less experienced readers and across centres with variable CMR expertise remains to be formally evaluated. However, the structured and rule-based nature of the method, combined with the availability of an online tool and a dedicated HTML calculator, may facilitate standardized application even among operators with limited experience in LGE quantification, provided that basic competence in LGE interpretation is ensured.

Beyond accuracy and reproducibility, VISTAQ demonstrated a dramatic reduction in analysis time. Median processing time was 105 s, compared with 375 s for conventional methods. This ∼70% reduction substantially improves workflow efficiency. Time efficiency is often overlooked in methodological studies but is critical for clinical adoption. A technique that is both highly reproducible and time-efficient is far more likely to be implemented in routine practice, particularly in high-volume imaging centres. Shorter analysis time may also reduce operator fatigue, indirectly contributing to further consistency.

### Limitations

Several limitations should be acknowledged. First, we compared VISTAQ with conventional LGE quantification methods; however, a true gold-standard reference technique is not available. As highlighted in previous studies,^4^ this represents an intrinsic limitation that cannot be fully overcome. Post-mortem validation in animal models is inadequate due to substantial differences between human and experimental infarction patterns, the absence of reliable animal models of adult sarcomeric HCM and myocarditis, and methodological constraints inherent to autopsy studies. These include the use of non-gated (or simulated gating) imaging protocols and the impossibility of performing true post-mortem LGE imaging, which limit the comparability with *in vivo* clinical CMR acquisitions.

Second, although we assessed both intra- and inter-observer reproducibility, we did not evaluate inter-scan reproducibility. Additional studies specifically designed to assess scan–rescan variability are warranted to further establish the robustness of the technique.

Third, the number of hard clinical events in the prognostic analysis was relatively limited. This may have led to inflation of HR estimates and widening of CIs, potentially affecting the precision of risk estimates.

Fourth, although the >10% LGE threshold is consistent with prior literature, prospective studies are needed to validate the optimal prognostic cut-offs when LGE is quantified using VISTAQ.

Fifth, the >10% LGE extent threshold used for prognostic stratification was identified by ROC analysis and subsequently applied for survival analysis within the same HCM cohort, without a separate validation set. This approach may be subject to optimism bias. Internal validation techniques, such as bootstrap resampling of the ROC analysis with bias-corrected CIs, or repeated *k*-fold cross-validation of the threshold performance, were not performed. Although the same >10% cut-off has been independently reported in prior studies using different quantification methods,^12–14^ which provides indirect external support for its clinical relevance. Prospective validation in independent, adequately powered cohorts is warranted before this threshold can be recommended for clinical decision-making.

Additional inherent limitations include the retrospective design with potential selection bias, the semi-quantitative nature of visual scoring which may have reduced spatial precision compared with voxel-based techniques, and a possible lower sensitivity for very small focal scars or insertion-point fibrosis that do not reach the threshold for mini-segment positivity.

## Conclusions

VISTAQ provides highly reproducible, time-efficient LGE quantification across multiple cardiac conditions and demonstrates a non-inferior prognostic performance in HCM compared with conventional threshold-based methods. If validated in larger and multicentre cohorts, VISTAQ has the potential to improve standardization of LGE assessment and refine arrhythmic risk stratification in clinical practice.

## Supplementary Material

qyag148_Supplementary_Data

## Data Availability

The data underlying this article will be shared by the corresponding author upon reasonable request.

## Lead author biography

Prof. Giovanni Donato Aquaro is an associate professor at the Department of Surgical, Medical and Molecular Pathology and Critical Care Medicine, University of Pisa, Italy. He completed his specialization in Cardiology at the University of Genoa and subsequently specialized in Radiology at the University of Pisa. From 2003 to 2008, he worked at the Institute of Clinical Physiology of the Italian National Research Council in Pisa. Between 2008 and 2022, he served at Fondazione Toscana Gabriele Monasterio. Since 2022, he has been Associate Professor at the University of Pisa. His main research interests include cardiac imaging, particularly cardiovascular magnetic resonance. He has authored over 300 scientific publications in cardiac magnetic resonance.
