# Supplementary material for: Visual standardized quantification of late gadolinium enhancement, a contour-less method for late gadolinium enhancement quantification
Source: Eur Heart J Imaging Methods Pract. 2026 Aug 26;4(3):qyag148. doi: 10.1093/ehjimp/qyag148 (PMC13594973; doi:10.1093/ehjimp/qyag148)
Supplement: Supplementary file 1 [file qyag148_supplementary_data.zip › supplemental figure captions.docx]

supplemental figure 1
Flowchart of patients enrolment for the reproducibility/comparison study

supplemental figure 2
Flowchart of patients enrolment for the prognostic study in a group of patients with Hypertrophic cardiomyopathy  
