# Supplementary material for: Visual standardized quantification of late gadolinium enhancement, a contour-less method for late gadolinium enhancement quantification
Source: Eur Heart J Imaging Methods Pract. 2026 Aug 26;4(3):qyag148. doi: 10.1093/ehjimp/qyag148 (PMC13594973; doi:10.1093/ehjimp/qyag148)
Supplement: Supplementary file 1 [file qyag148_supplementary_data.zip › VISTAQ.html.html]

LGE – VISTAQ (offline)


Click/tap to select. Right-click (desktop) or ‘Exclude radiobutton’ mode (mobile) to exclude/re-include a minisegment (LVOT).

# VISUAL STANDARDIZED QUANTIFICATION OF LATE GADOLINIUM ENHANCEMENT

# (VISTAQ-LGE method)

Number of basal slices:
Number of media slices:
Number of distal slices:
Generate

Select/deselect (white ↔ gray)


Exclude/include (black ↔ gray)

**N.of Macro-segments:**

**N. of Macro-segments with LGE:**

**N. of Mini-segments included:**

**N. of Mini-segments with LGE:**

**N. of Excluded Mini-segments:**

**LGE extent (%):** %
